# Selective Extrauterine Placental Perfusion in Monochorionic Twins Is Feasible—A Case Series

**DOI:** 10.3390/children11101256

**Published:** 2024-10-17

**Authors:** Benjamin Kuehne, Jan Trieschmann, Sarina Kim Butzer, Katrin Mehler, Ingo Gottschalk, Angela Kribs, André Oberthuer

**Affiliations:** 1 Division of Neonatology, Department of Pediatrics, Faculty of Medicine and University Hospital Cologne, University of Cologne, 50937 Cologne, Germany; 2 Department of Gynecology and Obstetrics, Faculty of Medicine and University Hospital Cologne, University of Cologne, 50937 Cologne, Germany

**Keywords:** delayed cord clamping, physiological based cord clamping, monochorionic twins, twin-to-twin transfusion syndrome, twin anemia-polycythemia sequence, very low birth weight infants

## Abstract

Background: Monochorionic (MC) twins are at risk for severe twin-to-twin transfusion syndrome (TTTS) or twin anemia-polycythemia sequence (TAPS). In the case of preterm delivery, cesarean section (CS) with immediate umbilical cord clamping (ICC) of both twins is usually performed. While the recipient is at risk for polycythemia and may benefit from ICC, this procedure may result in aggravation of anemia with increased morbidity in the anemic donor. The purpose of this study was to demonstrate that the novel approach of selective extrauterine placental perfusion (EPP) with delayed umbilical cord clamping (DCC) in the donor infant is feasible in neonatal resuscitation of MC twins and may prevent severe anemia in donor and polycythemia in the recipient. Methods: Preterm MC twins with antenatal suspected severe anemia of the donor as measured by Doppler ultrasound, born with birthweights < 1500 g by CS, were transferred to the neonatal resuscitation unit with placenta and intact umbilical cords. In the donor, the umbilical cord was left intact to provide DCC with parallel respiratory support (EPP approach), while the cord of the recipient was clamped immediately after identification. Results: Selective EPP was performed in three cases of MC twins with TAPS and acute peripartum TTTS. All donor twins had initial hemoglobin levels ≥ 13.0 g/dL, and none of them required red blood cell transfusion on the first day after birth. Conclusions: Selective EPP may be a feasible strategy for neonatal resuscitation of MC preterm twins with high stage TAPS and TTTS to prevent anemia-related morbidities and may improve infant outcome.

## 1. Introduction

Monochorionic (MC) twin pregnancies are associated with higher morbidity and mortality than dichorionic (DC) twin pregnancies and singletons [1,2], primarily due to placental vascular anastomoses that allow blood flow between the fetuses. Imbalanced flow can result in acute peripartum twin-to-twin transfusion syndrome (TTTS) or twin anemia-polycythemia sequence (TAPS) [3]. Both describe a chronic inter-twin blood transfusion leading to severe anemia in the donor and massive volume overload and polycythemia in the acceptor twin with (TTTS) or without (TAPS) oligo-polyhydramnios. [4,5,6]. Prenatal ultrasound assessment of middle cerebral artery (MCA) peak systolic velocity (PSV) allows reliable detection of anemia in MC twins, and a fetal intertwin MCA-PSV difference of >0.5 multiples of the median (MoM) has the greatest diagnostic accuracy for predicting TAPS and neonatal intertwin hemoglobin concentration difference [6,7,8].

The challenges with MC twins in neonatal resuscitation are that delayed umbilical cord clamping (DCC) is generally contraindicated because of the risk of increasing acute inter-twin transfusion [9]. Additionally, most cases of acute peripartum TTTS and TAPS are delivered by emergent cesarean section (CS), in which it may be difficult to differentiate between donor and recipient twins immediately after delivery during cesarean surgery. Thus, the physiological sequence of cord clamping after lung aeration cannot be maintained in the infants, as both cords are clamped immediately after birth, which can cause disturbances in cerebral blood flow and may increase the risk of neonatal complications, especially in preterm infants [10]. During the neonatal transition, anemia in the donor twin may be further exacerbated because aeration of the lungs entails an increase in pulmonary blood flow, resulting in a decreased left ventricular preload if there is no compensation from the intact placental circulation for the blood that is passing through the pulmonary circuit [11].

A possible approach to prevent aggravation of anemia in the donor in the case of severe peripartum TTTS or TAPS could be the extrauterine placental perfusion (EPP) approach, which allows a physiological sequence of lung aeration prior to cord clamping while the infant receives respiratory support by mask CPAP [12], even in the case of twin births. It has been shown to be feasible in preterm singletons and dichorionic twins [13].

In this study, we aimed to demonstrate the feasibility of a selective EPP with DCC exclusively in the donor infant during the neonatal resuscitation of MC twins with higher stage TTTS or TAPS and suspected anemic donor.

## 2. Patients and Methods

### 2.1. Patients

MC twin infants born with very low birth weight (<1500 g) between June 2017 and June 2019 and diagnosed with TAPS or severe peripartum TTTS were selected for this feasibility study in the perinatal tertiary care center of the University Hospital of Cologne, Germany. TTTS was diagnosed antenatally by ultrasound findings according to the Quintero staging system [14]: only poly-/oligohydramnios (Stage I), plus empty bladder in the donor (Stage II), plus critically abnormal Doppler studies including absent/reverse end-diastolic velocity in the umbilical artery, reverse flow in the ductus venosus, or pulsatile flow in the umbilical vein (Stage III) or plus hydrops (Stage IV). TAPS was also diagnosed prenatally according to the criteria by Tollenaar et al. [6]: middle cerebral arterial (MCA) peak systolic velocity (PSV) donor > 1.5 multiples of the median (MoM) and MCA-PSV recipient < 1.0 MoM (Stage 1), MCA-PSC donor > 1.7 and MCA-PSV recipient < 0.8 MoM (Stage 2), plus critically abnormal flow in donor (Stage 3), plus hydrops of donor (Stage 4). All clinical data were collected from the medical records.

During prenatal counseling, the EPP procedure was explained to the infants’ parents. Because selective EPP is not established for monochorionic pregnancies, informed consent was obtained from the parents of all infants included in this study.

The EPP procedure was videotaped in one case. The parents of the patients gave written informed consent before the recording. The study was conducted in accordance with the Declaration of Helsinki, and approved by the Ethics Committee of the medical faculty of the University of Cologne (Z. 24-1345).

### 2.2. Treatment

A CS was performed and both twins were delivered one after the other with their umbilical cords intact. The placenta was then gently detached by the obstetrician, and the twins were transferred to the neonatal resuscitation unit together with the placenta by the midwife. Resuscitation of both infants was performed according to our local protocol with immediate initiation of mask CPAP using the Benveniste valve with a stepwise increasing PEEP procedure, starting with CPAP levels of 8 cm H_2_O [15,16]. FiO_2_ levels were initially set at 0.21–0.3 and adjusted to maintain infants’ SpO_2_ levels at >10th percentile of the SpO_2_ values published by Dawson and colleagues for infants < 32 weeks’ gestation [17].

However, selective shortened cord clamping (30–60 seconds after birth) was performed in the recipient twins, whereas the donor twins were identified by pale skin color. In contrast, donor twins received EPP by holding the placenta ~40–50 cm above the infant’s heart level for several minutes while continuing to provide respiratory support by mask CPAP [13]. The umbilical cords of the donor infants were clamped and cut when they showed a regular respiratory pattern, stable heart rates > 100 bpm and an increasing pulse oxygen saturation (according to reference range by Dawson et al. [17]). All infants received early intravenous caffeine within 30 min of birth and less invasive surfactant administration (LISA) within the first 60 min of life according to our protocol [18].

## 3. Results

A total of 3 MC twin births with 6 infants were included in the study (Table 1):

### 3.1. Case 1

**Figure 1:**
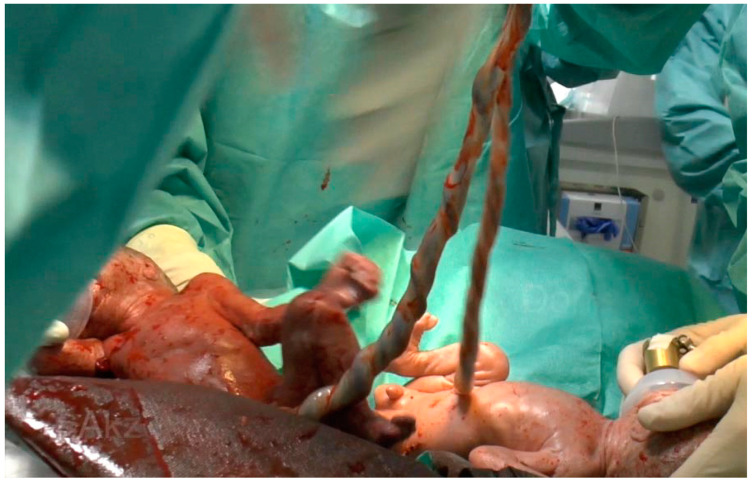
MC twins of Case 1 immediately after CS with umbilical cords still connected to the placenta. Respiratory support by mask CPAP is already initiated.

The pregnancy was uneventful after spontaneous conception until 18 weeks of gestation when ultrasound findings revealed a stage I TTTS. At 27 + 4 weeks’ gestation, a spontaneous TAPS was detected by Doppler ultrasound with an increased MCA-PSV of 66 cm/s (MoM 1.823) in the donor and MCA-PSV 30 cm/s (MoM 0.829) in the recipient. A resulting abnormal cardiotocographic (CTG) pattern in the donor resulted in preterm delivery at 27 + 6 weeks’ gestation. The twins were transferred to the resuscitation unit and the neonatal team started respiratory support with mask CPAP in both infants (Figure 1).

**Figure 2:**
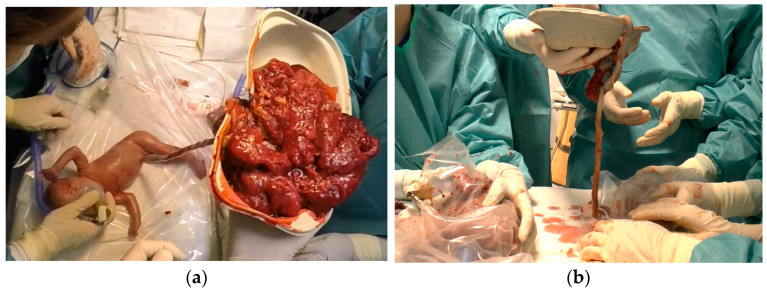
(**a**) Donor infant of Case 1 receiving a selective EPP procedure; (**b**) recipient (**left**) and donor infant (**right**) of Case 1 during neonatal transition 5 min after birth.

The recipient infant was identified within 30 s after birth and its umbilical cord was then immediately clamped, while the donor infant received an EPP procedure (Figure 2a,b) with a DCC of 7 min.

Both infants had a stable neonatal transition (Table 2). Initial hemoglobin (Hb) was 13.0 g/dL in the donor and 20.0 g/dL for the recipient. After admission to the neonatal intensive care unit (NICU), the donor received red blood cell transfusions on day 2 (Hb 9.0 g/dL) and day 29 (Hb 6.6 g/dL) of life.

Although the recipient’s umbilical cord was clamped immediately, isovolemic hemodilution was performed on the first day of life because the infant had severe polycythemia (Hb 25.0 g/dL) with signs of hyperviscosity (lactatemia > 3.0 mmol/L and reduced diuresis of <0.5 mL per kg body weight per hour). Both infants survived without major neonatal complications (Table 2) and were discharged from the hospital at a corrected age of 37 + 0 weeks of gestation (65 days of life).

### 3.2. Case 2

This twin pregnancy conceived by intracytoplasmic sperm injection (ICSI) was found to have a stage II TTTS by ultrasound at 21 weeks’ gestation. As the pregnancy progressed, ultrasound findings revealed both a stage III TTTS in the first infant and a selective intrauterine growth restriction (sIUGR) type III (according to the classification of Valsky et al. [19]) in the second infant. The mother was hospitalized at 26 + 5 weeks’ gestation for increasing polyhydramnios in one infant. At 27 + 4 weeks of gestation, Doppler ultrasound revealed an MCA-PSV of 61 cm/s (MoM 1.685) in the donor and an MCA-PSV of 41 cm/s (MoM 1.13) in the recipient. Emergent CS was performed for bradycardia in the donor infant on the same day. Twin infants were transferred together to the neonatal resuscitation unit and the neonatal team started the same procedures as described in Case 1 with selective EPP of the donor twin for 9 min, while the recipient’s umbilical cord was clamped immediately after identification approximately 30 s after birth.

The recipient infant had a stable neonatal transition; transition of the donor was compromised by pulmonary hypertension. Successful stabilization of the infant was achieved at 15 min of age with application of high oxygen levels (FiO_2_ 1.0) and inhaled Iloprost (synthetic analogue of prostacyclin PGI2). Both infants were transferred to the NICU, breathing spontaneously on CPAP. Initial Hb was 19.5 g/dL in the donor and 18.5 g/dL in the recipient. The donor received a red blood cell transfusion on day 5 of life (Hb 9.6 g/dL); the recipient did not require red blood cells during hospitalization. The recipient infant presented with hypertrophic cardiomyopathy during the first days after birth and required medical inotropic support. He had to be intubated on the third day of life due to respiratory failure and required invasive mechanical ventilation for 8 days. Cerebral ultrasound revealed a perinatal partial infarction of the right cerebral media area. Both the recipient and donor infant developed IV° retinopathy of prematurity (ROP) and received intravitreal anti-VEGF therapy. However, the donor infant had otherwise an unremarkable clinical course. Both infants were discharged from the hospital at corrected age of 39 + 6 weeks of gestation (87 days of life).

### 3.3. Case 3

After a normal twin pregnancy, ultrasound at 20 + 2 weeks gestation showed TTTS stage 1. Another Doppler ultrasound at 29 + 3 weeks’ gestation showed an MCA-PSV of 37 cm/s (MoM 0.937) in the recipient twin, while highly pathologic umbilical artery perfusion (absent of end-diastolic flow; TTTS stage 3) was detected in the donor twin. Notably, prenatal ultrasound revealed no donor/recipient weight differences. Measurement of MCA-PSV in the donor twin could not be performed as an immediate emergent delivery by CS was initiated. After transfer from the theater, postnatal identification of recipient and donor was hampered, because both infants presented with pale skin and only slight weight differences. Therefore, the cord of the recipient was clamped with a delay (30 s) after the infant was identified. The donor cord was clamped after 8 min of modified EPT procedure. After application of intravenous caffeine, both infants received LISA at 35 min (donor) and 45 min (recipient) without complications and were admitted to the NICU with CPAP respiratory support.

Initial Hb levels were 15.5 g/dL for the donor and 15.5 g/dL for the recipient. The further clinical course of both infants was unremarkable with no major neonatal complications (Table 2) and both were discharged from the hospital at a corrected gestational age of 364/7 (51 days of life).

## 4. Discussion

MC twins are at risk for severe complications such as TTTS and TAPS, which are challenging for both obstetricians and neonatologists. Most TTTS infants are born prematurely and require respiratory support during neonatal resuscitation. In this classical theater setting, it is not possible for obstetricians to perform DCC exclusively for the donor [20], resulting in most cases of ICC for both twins. In our study, we were able to demonstrate for the first time that the procedure of EPP is feasible in MC twins, allowing exclusive DCC for the donor infant while avoiding overloading the recipient’s circulation by shortened umbilical cord clamping.

Furthermore, DCC with simultaneous respiratory support of the donor in the case of preterm delivery could be beneficial for several reasons: First, DCC has become standard practice to support the neonatal transition of preterm infants, as it has been shown to improve survival [21], which may be partly related to prolonged placental transfusion with better hemodynamic stability during the process of fetal to neonatal transition [22]. On the other hand, clamping the umbilical cord after lung aeration is beneficial because placental blood supply during lung aeration improves cardiac output and pulmonary blood flow. In the case of an ICC before lung aeration, there may not enough blood available to compensate for the increased pulmonary blood flow. This might jeopardize premature infants in whom low cardiac output due to inadequate preload might coincide with hypoxia due to lung immaturity, leading to a high risk of developing a pulmonary hypertension which is one of the most serious complications of TTTS [23]. Prolonged placental transfusion until the lungs are aerated may protect against such a scenario—a procedure termed physiologically based cord clamping (PBCC). It is important to emphasize that this physiological process of successful lung aeration is not necessarily equivalent to the infant’s respiratory effort and extends to the end of the neonatal transition—a process that requires prolonged placental perfusion for several minutes, especially in immature preterm infants [24].

Recently, we demonstrated that PBCC by using the EPP approach is feasible in singletons and DC twins and may improve oxygenation during neonatal transition [13]. In particular, preterm twins may benefit from PBCC, as they are more likely to have unfavorable outcomes compared to singletons born at similar gestational ages. While DCC in preterm DC twins has been shown to be safe and may be associated with higher postnatal Hb levels [25] and a reduction in early red blood cell transfusion [26], evidence for DCC in MC twins is limited [20,27], which may reflect the complexity of umbilical cord clamping in neonatal resuscitation of MC twins.

However, the majority of donor twins in TTTS do require early red blood cell transfusion [4]. Lewi et al. described the Hb level in anemic twins as <11 g/dL in the first TAPS definition, whereas a level of >20 g/dL is present in the polycythemic co-twin is present [28]. All donor infants in our case series had initial hemoglobin levels ≥ 13.0 g/dL, highlighting the potential benefit of selective EPP. For the EPP procedure, we have shown an estimated placental blood transfusion of 20 mL/kg body weight, which represents 20–25% of total potential blood volume at birth [13,29].

The study is limited by the fact that we describe only a few cases of severe TTTS and TAPS MC infants. In addition, all attending obstetricians and neonatologists at our center are experienced in EPP and can perform delivery of both twins with subsequent delivery of the placenta. Therefore, our observations cannot be generalized to other settings and centers. In addition, there was no control group of MC infants who were resuscitated without selective EPP, and potential sources of bias were not considered. Further controlled multicenter studies comprising a larger cohort are needed to evaluate the effects of EPP on hematologic and other neonatal short- and long-term outcomes in twins as well as the potential harms of this specific procedure to the infants. Due to the rare nature of these events, it must be considered challenging.

## 5. Conclusions

We conclude that a selective EPP is feasible for supporting the neonatal transition of MC preterm twins. It represents, in our considered opinion, the only available strategy for the postnatal management of severe TAPS and TTTS with acute peripartum anemia to prevent severe anemia-related morbidities and may improve neonatal outcome.

## Figures and Tables

**Table 1:** Basis data.

	Case 1 (Donor/Recipient)	Case 2 (Donor/Recipient)	Case 3 (Donor/Recipient)
Gestational age, wk + days	27 + 6	26 + 6	29 + 3
Birth weight, g	980/1220	728/950	1170/1270
Sex, female (f)/male (m)	m/m	f/f	m/m
Primipara (y/n)	y	n	n
Maternal age, yrs	37	41	37
Antenatal corticosteroids (y/n)	y	y	n

**Table 2:** Neonatal outcome.

	Case 1 (Donor/Recipient)	Case 2 (Donor/Recipient)	Case 3 (Donor/Recipient)
APGAR scores (1′,5′,10′) Time of umbilical cord clamping, s Early caffeine administration	6,8,9/7,9,9	1,4,7/7,8,8	7,8,9/7,8,9
	420/≤30	540/≤30	480/30
	+/+	+/+	+/+
LISA Respiratory support in delivery room	+/+	+/+	+/+
		
nCPAP NIPPV Intubation and mechanical ventilation Mean temperature at admission (°C)	+/+	+/+	+/+
	−/−	−/−	−/−
	−/−	−/−	−/−
	36.5/36.8	37.3/36.9	36.8/36.0
Mean hematocrit (hemoglobin) in the first 24 h of life (%/(g/dL)) Red blood cell transfusions during first 7 days, No.	40 (11.0)/70 (21.0)	62.5 (19.5)/60 (18.5)	52.5 (16.25)/50 (15.25)
	1/0	1/0	0/0
Red blood cell transfusions during first 28 days, No.	2/0	1/0	0/0
Invasive mechanical ventilation on NICU	−/−	−/+ (after 72 h of life)	−/−
IVH, all grades	−/−	−/− (right parieto-occipital cerebral ischemia)	I°/I°
BPD (moderate/severe)	−/−	−/−	−/−
NEC/SIP	−/−	−/−	−/−
ROP (grade)	−/−	IV° with Plus and Bevazicumab treatment/IV° and Bevazicumab treatment	−/−

## Data Availability

The original contributions presented in the study are included in the article.
